# Ovarian response and conception rate in Boran and Boran*Holstein cows treated by Gonadotrophin-realizing hormone and ProstaglandinF2α with and without exogenous progesterone

**DOI:** 10.1186/s12917-023-03617-0

**Published:** 2023-06-01

**Authors:** Tilaye Demissie Ayanie, Alebachew Tilahun Wassie, Ebisa Merga Kebede, Tefera Yilma Mekonnen, Tamrat Degefa Geleto, Alemayehu Lemma Biru

**Affiliations:** 1 College of Veterinary Medicine & Agriculture, Addis Ababa University, P.O. Box 34, Bishoftu, Ethiopia; 2 School of Animal Science and Veterinary Medicine, Bahir Dar University, Bahir Dar, Ethiopia; 3 Ministry of Agriculture, Addis Ababa, Ethiopia; 4 Institute of Agricultural Research, Debre-Zeit Agricultural Research Center, Bishoftu, Ethiopia

**Keywords:** Boran, Boran*Holstein, Breed, Conception rate

## Abstract

**Background:**

Difference in breed, nutrition status and climate in which animals are managed result in differences in response to reproductive hormones. Fertility rate to artificial insemination is very low in Ethiopian Boran and Boran*Holstein crosses. This partly maybe due to adopting estrus and/or ovulation synchronization developed for temperate taurine cattle. Experimental study was conducted to evaluate ovarian response to combinations of Gonadotrophin-Realizing Hormone agonist (gonadorelin) and ProstaglandinF2α (PGF2α) with or without progesterone (Controlled Internal Drug Release/CIDR), and conception rate to timed AI. Postpartum native Ethiopian Boran (n = 60) and Boran*Holstein cross (n = 66) cows were randomly assigned to four treatment groups as Ovsynch (gonadorelin on day of start, PGF2α seven days later, 2nd gonadorelin at 48 h of PGF2α and insemination at 19 h of the 2nd gonadorelin); CIDR + Ovsynch (same as Ovsynch but CIDR device was inserted into vagina for 7 days); Cosynch (same as Ovsynch but insemination was made at the 2nd gonadorelin) and CIDR + Cosynch (same as Cosynch but CIDR was inserted for 7 days).

**Result:**

There was no difference (P > 0.05) in ovulation rate to day 9 gonadorelin (88.33% in Boran; 78.79% in Boran*Holstein) and interval from day 9 gonadorelin to ovulation (36.5 ± 1.13 h in Boran and 36.057 ± 1.11 h in Boran*Holstein). Dominant follicle immediate to ovulation (14.95 ± 0.19 mm Vs 19.12 ± 0.49 mm) and corpus luteum size (16.31 ± 0.33 mm Vs 20.28 ± 0.43 mm ) respectively were smaller (P < 0.05) in Boran than Boran*Holstein. Plasma progesterone concentration at PGF2α was higher (P < 0.05) in Boran (11.91 ± 0.74ng/mL) than Boran*Holstein (6.13 ± 0.27ng/mL) but luteolysis rate was lower (P < 0.05) in Boran (87.9%) than Boran-Holstein (96.9%). Cows with CIDR had higher conception rate than cows without CIDR (72.00% Vs 39.02% in Boran*Holstein and 74.07%, Vs 51.52% in Boran respectively). Insemination at 19 h of gonadorelin administration resulted in higher conception rate (78.6% for Boran; 71.43% for Boran*Holstein) than insemination at gonadorelin (69.29% for Boran; 66.67% for Boran*Holstein).

**Conclusion:**

Boran cows have smaller preovulatory follicles, smaller corpus luteum, large amount of progesterone and lower rate of luteolysis to PGF2α compared to Boran*Holstein. The CL of Boran cattle seems les reactive to PGF2α than Boran*Holstein CL. CIDR significantly improved conception rate in Boran and Boran*Holstein cows.

## Introduction

Boran cattle are the dominant cattle breed, most popular as beef breed in eastern African countries such as Ethiopia, Kenya, Tanzenia, Uganda and Zambia. The Ethiopian Boran, a dual purpose breed, is one of the cattle breeds widely used in Ethiopia. The breed is well adapted to arid and semi-arid tropical conditions, is tolerant to many of prevailing diseases [1]. In Ethiopia Boran cows are used for crossbreeding with Holstein semen to produce crossbred heifers for milk production.

Fertility rate to estrus synchronization and AI are low in zebu and zebu*Holstein crosses in Ethiopia. This partly maybe due to adopting estrus and/or ovulation synchronization developed for temperate taurine cattle. In zebu cattle estrus is short, most estrus are manifested during night, most mountings are not accepted and some animals are characterized by few mounts and these traits make it difficult for traditional estrus detection. Many ovulation synchronization protocols have been tested and found effective; however, nearly all of them were developed for *Bos taurus* cattle reared in either temperate or humid tropical climate. Almost all reports on ovarian physiology and response of *Bos indicus* to reproductive hormone treatments were from Brahman, Nelore and Gir breeds [13–16, 19, 20]. However, ovarian physiology and response to reproductive hormone treatment vary depending on breed, nutritional status and climatic region in which animals are managed.

Pursley et al.[2] developed effective ovulation synchronization protocol that uses administration of GnRH followed 7 day later with PGF2α, GnRH 48 h after PGF2α and insemination at 16 h of GnRH. This ovulation synchronization protocol has been modified several times and now is in use with pregnancy rate that gives equivalent to insemination at estrus detection. When this protocol is used in cattle of *Bos indicus* breed the pregnancy rate is approximately 30% [3]. There is no information on protocol that utilizes GnRH and PFG2α either with or without progesterone in cattle of *Bos indicus* and their cross with *Bos taurus* that are reared in African climatic environment. The objectives of the present study were to evaluate ovarian follicular and ovulatory response to exogenous reproductive hormones; and to assess hormone combination that give better conception rate in Boran and Boran-Holstein cattle.

## Materials and methods

The study was conducted in Arsi University which is located in Asella town. Asella town is located about 175 km Southeast of Addis Ababa, the capital of Ethiopia, at 6° 59’ to 8° 49’ N latitude and 38° 41’ to 40° 44’ E longitude. The altitude of the area ranges from 2500 to 3000 m.a.s.l. The minimum and maximum temperature ranges from 8.4 to 22.6 °C, and the relative humidity ranging from 43 to 60%. The average rainfall is 2000 mm [4].

### Animals management

The study was conducted on cows of Arsi University which were maintained for teaching and research purpose. The authors’ had received consent to use the cows after officially applied to College of Agriculture and Natural resource of Arsi University and all procedures were carried out in accordance with ARRIVE guidelines. The study animals were parity one Boran (n = 60) with 3.69 ± 0.75 years age and parity one Boran*Holstein (BH) crossbred cows (n = 66) with 3.79 ± 0.18 years. The cows were housed in free-stall barns made of wood and concrete floor. Cows were free grazing and were supplemented with 3-4.5 kg roughages (dry grass hay) and 1.5-2.5 kg concentrates mix made of wheat bran, oil seed cakes and salt. Lactating cows were milked manually twice daily at an interval of 12 h. Water was *ad libitum*.

### Experimental design

**Fig. 1:**
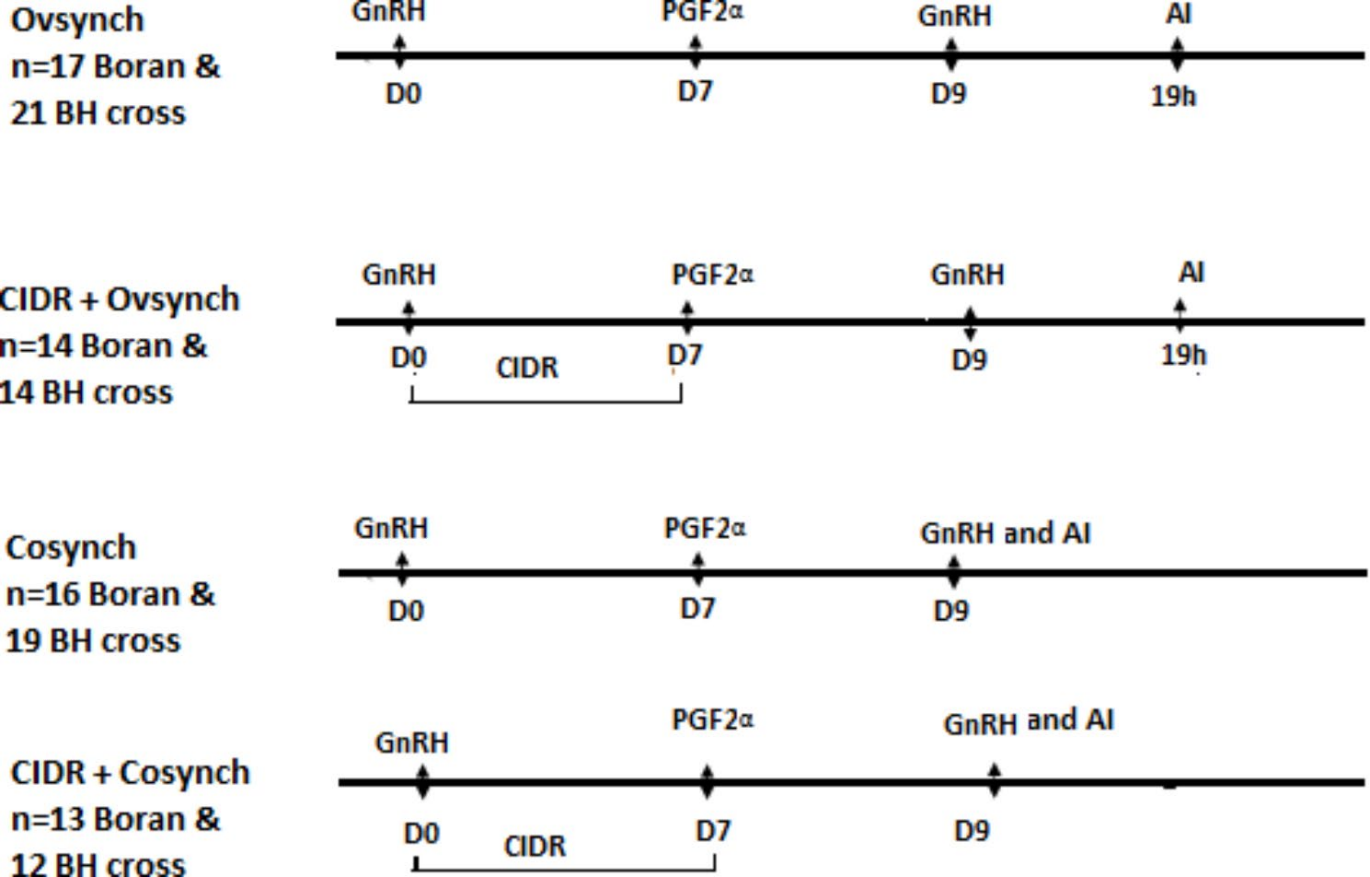
Pictorial representation of the experimental design. BH = Boran*Holstein cross cows

Cows were first blocked by breed into two and then within the breed, cows were randomly assigned to four treatments groups: The experimental design is indicated in Fig. 1. Briefly, group 1 cows (Boran = 17, BH = 21) received100µg gonadorelin (GnRH agonist, Gonadorelin diacetate tetrahydrate, Merial limited Duluth, USA) on starting day (D0). On seventh day (D7) all cows received 500 µg PGF2α (Synchromate®, cloprostenol sodium,Warburg, Germany). On the ninth day (D9) cows received a second 100 µg gonadorelin and the group was assigned as Ovsynch. Group 2 cows (Boran = 14, BH = 14) were treated as in Group1 (Ovsynch) but on D0 cows received intra-vaginal progesterone (CIDR 1380, EAZI BREED™, New Zealand). The CIDR was retained for 7 days and at CIDR removal (D7), PGF2α was given. The group was assigned as CIDR + Ovsynch. In group Ovsynch and CIDR-Ovsynch insemination was made at 19 h of D9 gonadorelin with frozen thawed semen. Group3 cows (Boran = 16, BH = 19) were treated as in Group1 but insemination was made at second gonadorelin administration and the group was assigned as (Cosynch). Group 4 cows (Boran = 13, BH = 12) were treated as in group 2 but insemination was made at the second gonadorelin and the group was assigned as CIDR + Cosynch. All injections were given IM into the gluteal muscle and all cows were inseminated by one technician and semen was from one bull.

### Ovarian ultrasonography

Mindray ultrasound system (DP.50vet, China) with a 7.5 MHz linear array rectal probe was used. In all cows ovaries were monitored on D0, D2, D7, D8, D9 and then after at 24 h, 36 and 48 h after D9 to assess ovulatory outcomes and size of ovulatory follicle. On ultrasongraphic examination, the size of follicles, the location of the dominant follicle and corpus luteum were recorded. Ovulation was confirmed on disappearance of a previously identified dominant follicle ≥ 8 mm and presence of CL on the same site [5]. Response to first GnRH injection was assessed by the development of a new CL on D7 regardless of their initial CL.9.

### Plasma P4 analysis

Blood samples were collected on D0, D7, and D9 and at AI from the jugular vein into vacuum tube with EDTA (ZheJiang Msedical Technology, China). After collection blood was immediately centrifuged at 3000 g for 15 min and the separated plasma was stored at -20^0^ C for future P4 analysis.

Samples were analyzed at Ethiopian Public Health Institute, using radioimmunoassay (RIA) kit (Roche Diagnostics Gmbh, Mannheim, Germany). The kit has lower and upper range of 0.010ng/ml and 60ng/ml, respectively. A concentration higher than 1 ng/ml was considered to indicate the presence of a functional CL. Corpus luteum regression was defined by a cow having plasma P4 concentration of > 1 ng/ml at PGF2α, and then declining P4 to a level of < 1 ng/ml within 48 h of PGF2α. A cow was assumed to be cycling if she had plasma progesterone ≥ 1ng/ml and visible CL at ultrasound at day of start or said be non-cycling if she had plasma progesterone ≤ 1ng/ml and no visible CL at ultrasound.

### Pregnancy determination

Conception was checked on D32. On ultrasound, the presence of fluid-filled uterine horn and presence of a conceptus were used as positive indicators of conception [6].

### Statistical analysis

The effects of breed, parity, cyclical status, BCS, CIDR insertion, insemination time, luteolysis rate, and treatment type on pregnancy rate were compared by logistic regression. Count data like intervals from PGF2α to ovulation or CIDR removal to ovulation, gonadorelin to ovulation, diameter of follicles at ultrasound, diameter of CL were compared either using analysis of variance (ANOVA) or Student *t* test in STATA software (Version 12). All count measurements were indicated as mean ± SE (standard error of the mean). *P* < 0.05 was considered to be significant. Conception rate was defined as the number of cows that became pregnant, divided by the number of cows that were inseminated.

## Results

### Ovarian follicular dynamics

The details of ovarian folliclar dynamics are indicated in Table 1. There was no difference in ovarian cyclicity (P > 0.05) at the beginning of the treatment (61.67% in Boran and 64.62% in Boran*Holstein). Dominant follicle size at application of the first gonadorelin was significantly smaller (P < 0.05) in Boran cows (9.5 ± 0.23) than in Boran *Holstein (13.00 ± 0.36). Similarly, dominant follicle at PGF2α, at second gonadorelin, and immediate to ovulation were smaller (P < 0.05) in Boran cows than in Boran*Holstein (Table 1).

**Table 1:** Ovulation rate to gonadorelin and variation in follicle size by breed

Items Measured	Breed		P. Value
	Boran	Boran*Holstein	
DF at D0 GnRH (mm)	9.50 ± 0.26	13.00 ± 0.36	0.00
DF at PGF2α (mm)	12.00 ± 1.64	15.18 ± 0.56	0.03
DF at 48 h of PGF2α (mm)	12.12 ± 0.18	15.81 ± 0.46	0.00
DF at 24 h of d9 GnRH (mm)	14.58 ± 0.17	17.55 ± 0.41	0.00
DF immediate to ovulation	14.95 ± 0.19	19.12 ± 0. 49	0.00
Ovulation to D0 gonadoreline (%, N0)*	48.33(29/60)	43.94(29/65)	0.621
Ovulation to D9 gonadoreline (%,N0)**	88.33 (53/60)	78.79(52/66)	0.15

DF (dominant follicle), *= cows with follicle ≥ 10 mm on D0 and CL on the same site 48 h latter, **= cows with follicle ≥ 8 mm on D7 or follicle ≥ 10 mm on D9 and Cl on the same site 48 h latter. Follicle diameter measurements were in mean ± SE.

The details of treatment * breed effect on follicle dynamics are indicated in Table 2. In both breeds, cows with CIDR insert had significantly larger (P < 0.05) dominant preovulatory follicles than cows without CIDR. Ovulation 48 h after D0 gonadorelin had no significant effect (P > 0.05) on preovulatory follicle size. The preovulatory follicle size was 15.06 ± 0.28 mm for Boran cows ovulated to D0 gonadorelin and 14.88 ± 0.27 mm for cows that do not ovulate. In Boran*Holstein preovulatory follicle size was 16.77 ± 0.29 mm for cow ovulated to D0 gonadorelin and 16.22 ± 0.27 mm for cows that do not ovulate.

### Ovulation rate and time of ovulation

When treatment protocol was not considered, the difference in overall ovulation rate to the D9 gonadorelin was not significant (P > 0.05) by breed (88.33% for Boran; 78.79% for Boran*Holstein) (Table 1). However, when breed and treatment protocol combined and compared, CIDR insertion significantly increased (P < 0.05) ovulation rate to the D9 gonadorelin in both breeds (Table 2). In both breeds, ovulation rate to the D9 gonadoreline was significantly affected (P < 0.05) by cycling status at the start of experiment with 16.7% of non-cycling and, 88.3% of cycling Boran and 21.5% of non-cycling and, 78.5% of cyclin Boran*Holstein cows were ovulated. Ovulation rate to the D9 gonadoreline was not affected (P > 0.05) by BCS and parity.

There was no difference (P > 0.05) on interval from D9 gonadoreline to ovulation by breed (36.5 ± 1.13 h for Boran; 36.057 ± 1.11 h for Boran*Holstei). However, the interval from CIDR removal to ovulation was tended to be longer (P = 0.08) in Boran (83.18 ± 1.12 h) than Boran*Holstein cows (77.57 ± 3.12 h). The distribution of time of ovulation was similar (P > 0.05) between Boran and Boran*Holstein cows. Thirty four Boran cows (64.2%) ovulated between 24 and 36 h of GnRH while 19 cows (35.8%) ovulated between 36 and 48 h of GnRH. In Boran*Holstein breed 29 cows (55.8%) ovulated between 24 and 36 h of GnRH while 23 cows (44.2%) ovulated between 36 and 48 h of gonadorelin.

**Table 2:** Follicular size and ovulation rate to gonadorelin by Breed*Treatment interaction

Item measured	Breed * Treatment protocol			
	Ovsynch Boran	Ovsynch + CIDR Boran	Ovsynch BH	Ovsynch + CIDR BH
DF at PGF2α	9.99 ± 0.29^**a**^	14.46 ± 3.60^**b**^	14.03 ± 3.60^**b**^	16.96 ± 0.90^**c**^
DF at 48 h of PGF2α	11.47 ± 0.22^**a**^	12.91 ± 0.24^**a**^	14.92 ± 0.56^**b**^	17.05 ± .73^**c**^
DF at 24 h of D9 gonadoreline	13.34 ± 0.78^**a**^	15.01 ± 0.20^**b**^	16.81 ± 0.49^**c**^	18.52 ± 0.66^**d**^
DF immediate to ovulation	14.11 ± 027^**a**^	15.36 ± 0.24^**b**^	18.29 ± .72^**c**^	20.20 ± .27^**d**^
Ovulation to D0 gonadorelin (%, N0)	48.5(16/33)^**a**^	48.2(13/27)^**a**^	42.5(17/40)^**b**^	46.2(12/26)^**a**^
Ovulation to D9 gonadorelin (%,N0)	84.9(28/33)a	92.6(25/27)b	77.5(31/40)c	80.8(21/26)d

DF (Dominant follicle), BH ( Boran*Holstein cross), Ovsynch (Ovulation synchronization), CIDR (Controlled Internal Drug Release), a, b, c, d (across the raw values with different scripts are significantly different at P < 0.05).

### Corpus luteum dynamics, luteolysis and plasma progesterone

The size of CL was significantly (P < 0.05) smaller for Boran (16.31 ± 0.33 mm) than Boran*Holstein (20.28 ± 0.43 mm) cows (Table 3). Plasma progesterone concentration at PGF2α was significantly higher (11.91 ± 0.74ng/mL) in Boran cows than Boran*Holstein (6.13 ± 0.27ng/mL).

**Table 3:** Corpus luteum size and plasma P4 by breed and breed*treatment protocol

Item measured	Breed		Breed * Treatment Protocol			
	Boran	BH	Ovsynch Boran	Ovsynch + CIDR Boran	Ovsynch BH	Ovsynch + CIDR BH
CL Size(mm)	16.31 ± 0.33	20.28 ± 0.42	15.83 ± 0.44	16.98 ± 0.48	20.11 ± 0.52	20.50 ± 1.0
Previous CL (%)	71.67	69.23	57.58	66.67	62.50	68.00
New CL (%) †	48.33	43.94	48.5	48.15	42.50	46.15
Total CL on d7 (%)	83.05	84.85	84.85	92.59	77.50	80.77
P4 (at PGF2α) (ng/ml)	11.97 ± 0.74^**a**^	6.76 ± 0.34^**b**^	6.8 ± .76^**a**^	13.83 ± 1.85^**b**^	5.24 ± .26^**a**^	9.04 ± .69^**b**^
P4 (at AI) (ng/ml)	0.58 ± 0.06	0.51 ± 0.06	0.58 ± 0.12	0.57 ± 0.13	0.46 ± 0.10	0.54 ± 0.13

BH (Boran*Holstein), CL (Corpus Luteum), † (All CL other than D0 CL), a, b (Consecutive values with superscript within the raw were significantly (P < 0.05) different), Ovsynch (ovulation synchronization), CIDR (Controlled Internal Drug Release).

CIDR insertion at the beginning of experiment increased proportion of cows with P4 concentration greater than 3ng/mL at PGF2α by 3.94% in Boran cows and by 12.3% in Boran*Holstein. In both breeds, cows with CIDR insert had significantly higher (P < 0.05) plasma P4 concentration at PGF2α than cows without CIDR inserts. Cows that ovulated to initial gonadoreline had significantly greater (P < 0.05) plasma P4 than cows that did not ovulated (Table 4). There was (P < 0.05) breed * ovulation status to initial gonadorelin effect on plasma P4 concentration at PGF2α with Boran cow ovulated to initial gonadoreline had higher plasma P4 concentration than Boran*Holstein cows ovulated to initial gonadorelin. Plasma P4 was significantly affected (P < 0.05) by cyclical status at the start of the experiment with cycling cows with higher P4 in both breeds (Table 4). The rate of luteolysis was significantly (P < 0.05) lower in Boran (87.9%) than Boran*Holstein cows (96.9%). However, the difference in the rate of lueolysis was not significant (P < 0.05) by CIDR insert (Table 3). Body condition score and parity did not affect (P > 0.05) plasma P4 concentration at PGF2α, at insemination (Table 4).

**Table 4:** Effect of different factors on plasma progesterone at PGF2α and at insemination

Breed	P4 Concentration	Parity		Body condition score		Ovulation to D0 GnRH		Cyclical status at D0	
**Boran**		Primiparous	Multiparous	2.75-3	> 3–5	Yes	No	Yes	No
	P4 at PGF2α	5.15 ± 0.19	5.45 ± 0.34	5.10 ± 0.21	5.49 ± 0.27	5.31 ± 0.28^**a**^	4.22 ± 0.32^**b**^	6.15 ± 1.23^**a**^	3.44 ± 0.83^**b**^
	P4 at AI	0 0.60 ± 0.09	0.51 ± 0.67	0.50 ± 0.60	0.61 ± 0.10	0.67 ± 0.13	0.58 ± 0.04	0.66 ± 0.08	0.72 ± 0.16
**Boran-Holstein**	P4 at PGF2α	6.31 ± 53	5.80 ± 0.23	7.71 ± 0.61	6.87 ± 0.1.79	8.12 ± 1.13^**a**^	5.82 ± 0.76^**b**^	7.71 ± 0.61	6.87 ± 1.97
	P4 at AI	0.69 ± 0.57	0.60 ± 0.87	0.65 ± 0.11	0.67 ± 0.69	0.54 ± 0.54	0.57 ± 0.20	0.53 ± 0.03	0.46 ± 0.33

a, b Consecutive values with superscript within the raw were significantly (P < 0.05) different

### Conception rate

Overall conception rate was not different (P > 0.05) by breed (66.7% in Boran and 53.0% in Boran*Holstein). In both breeds, CIDR insert had resulted in significantly higher (P < 0.05) conception rate irrespective of time of AI (Table 5). When time of insemination was considered, insemination19h after the second gonadoreline (Ovsynch) resulted in higher conception rate (P < 0.05) than insemination at gonadoreline (Cosynch ) but in a group of Borans without CIDR, Cosynch yield higher conception rate (P > 0.05) than Ovsynch. Boran cows that received CIDR insert and inseminated 19 h after the second gonadoreline had significantly higher (P < 0.05) conception rate than other groups. There was interaction of hormone protocol * time of insemination * breed effect on conception rate (P < 0.05) in which the lowest conception rates was recorded in Boran*Holstein cows that were ovulation synchronized without CIDR insertion and inseminated by Ovsynch and Cosynch protocol (Table 5).

**Table 5:** Effect of CIDR insert and insemination time on conception rate in the two breeds

Breed	Treatment protocol	Conception (%, N0)	OR	P. Value	[95% CI of OR]
**Boran cows**	Ovsynch	40.1(8/17)	Ref		
	CIDR + Ovsynch	78.6(11/14)	5.73	0.02	1.9392742 - 9.838907
	Cosynch	56.3(9/16)	1.58	0.33	0.4743466 - 9.296992
	CIDR + Cosynch	69.23(9/13)	2.10	0.04	1.098612 - 5.42229
Boran*Holstein **cows**	Ovsynch	42.86(9/21)	Ref		
	CIDR + Ovsynch	71.43(10/14)	4.70	0.01	2.473608 - 7.914793
	Cosynch	36.84(7/19)	0.41	0.38	0.181718–2.578733
	CIDR + Cosynch	66.67(8/12)	3.26	0.01	1.73039 - 10.66341

OR (Odds Ratio), Ref (Reference), CI (Confidence Interval), CIDR (Controlled Internal Drug Release, progesterone source), Ovsynch (Ovulation synchronization & insemination at 19 h of final gonadorelin), Cosynch (Ovulation synchronization & insemination at final gonadorelin).

In both breeds, irrespective of treatment protocol and time of insemination, conception rate was significantly higher (P < 0.05) in cows that were cycling at the beginning of the study (Table 6). In Boran*Holstein, it was found that parity significantly affected (P < 0.05) conception rate with primiparous cows having greater conception. In Boran cows the difference in conception rate was not significant although primiparous cows tend to have greater conception rate than multiparous (Table 6). In both breeds, conception rate was significantly higher (P < 0.05) in cows with body condition score > 3.

**Table 6:** Effect of breed, parity and body condition score on conception rate to timed artificial insemination

Factors considered	Breed				
	Boran			Boran*Holstein	
		Conception (%, N0)	P-Value	Conception (%, N0)	P. value
Parity	Primiparous	68.0(34/50)	0.06	55.6(30/54)	0.01
	Multiparous	60.0(6/10)		41.7(5/12)	
BCS	2.75 to 3	58.3(7/12)	0.04	47.6(10/21)	0.05
	> 3	68.8(33/48)		55.6(25/45)	
Cyclic stat	Cycling	72.1(31/43)	0.01	57.8(26/45)	0.03
	noncycling	52.9(9/17)		42.9(9/21)	
Overall conception		66.7(40/60)*		53.0(35/66)*	

BCS = Body condition score, * = difference in conception rate was not significant (P > 0.05)

## Discussion

Ovulation to initial (D0) and D9 gonadorelin was slightly higher (P > 0.05) in Boran (48.3% Vs 88.3%) than Boran-Holstein (43.9% Vs 78.8%) cows. The present ovulation to initial gonadorelin was higher than the 23%, and lower than the 54% and 96% ovulation in cattle of *Bos taurus* breed [7]. The difference in ovulation rate might be due to breed. Cow used in this study were *Bos indicus* (Boran) and *Bos indicus* influenced Holstein (Boran*Holstein). Bó et al. [8] indicated that cattle of *Bos indicus* have three or four wave ovarian follicle which increases the likelihood that fewer cow respond to the gonadorelin given at random stage of estrus cycle. The difference in ovulation rate might be due to management (nutrition), and stage of estrus cycle. Gonadorelin given at early stage of the estrus cycle leads to low ovulation rate as the follicles are small and do not express enough LH receptors. Similarly, when gonadorelin is given near mid cycle as there is loss of functional dominance in most large follicles of the first wave it would leads to decreased ovulation rate [9, 10].The low ovulation rate to gonadorelin might also be due to low response to gonadorelin of acycling cows.

The most probable reason ovarian follicle size at PGF2α, at gonadorelin and at ovulation were significantly larger (P < 0.05) in Boran*Holstein than Boran was the modification by taurine blood. Previous studies indicated that ovarian follicle were larger in *Bos taurus* [11] even at follicular deviation stage than *Bos indicus* [12–14].

In present study, in Boran (*Bos indicus*) ovulation was not occurred at follicle size less than 10 mm. However, Gimenes et al. [12] using exogenous LH reported that *Bos indicus* heifers can ovulate from follicles that were 7 -10 mm. The ovulatory follicle size (16.0 ± 0.18 mm) in Boran in this study was larger than the range (11 to 14 mm) previously reported from Nelore [13, 15, 16]. The ovulatory follicle size (19.6 ± 0.53 mm) in Boran * Holstein cows was within the size range (13 to 19 mm) of Holstein cattle [17, 18]. An increase in ovulatory follicle size in cross breed cattle is most probably a modification by taurine blood.

Cows breed significantly affected CL in present study. The CL size (16.31 ± 0.33 mm) was significantly smaller (P < 0.0) in Boran than Boran * Holstein cows (20.28 ± 0.42). Similarly, Boran CL size was smaller than the CL range (17 to 21 mm) for other zebu cattle [15, 19]. The CL size of Boran * Holstein cows was also smaller than the CL range (20 to30mm) for Bos taurus cattle [5]. In addition to breed some other inherent factors and nutrition may affect CL size. In 22 Boran and 17 Boran * Holstein cows the CL that were present at day of start were lost at PGF2α and may be that the Ovsynch was initiated at late diestrus which often leads to premature regression of CL.

The plasma P4 (11.91 ± 0.74ng/mL) in Boran in the present study was higher than previous report on *Bos indicus* (Nelore) cows [15, 20]. Similarly higher plasma P4 in Boran than Boran * Holstein cows in this study was consistent with that of Carvalho et al. [21] who compared *Bos indicus* (Nelore & Gir) with crossbreds (Angus * Nelure & Gir * Holstein) and reported higher plasma P4 in *Bos indicus*. The difference in plasma P4 might be explained by differences in nutrition as studies indicated lesser circulating steroid hormones in animals with greater feed intake [22].

The rate of luteolysis in Ovsynch group in present study was lower than the 84.8% previously reported from Holstein cows [23]. The difference might be due to breed difference or it might be due to day of estrus cycle at which experiment started. Moreira et al. [10] indicated that plasma P4 start to decrease prior to injection of PGF2α when Ovsynch was started at 15th day of estrus cycle. In line with the present finding, previous works Carvalho et al.[21] and Lima et al. [24] indicated that cows with CIDR insert had greater plasma P4 than cows without CIDR insert.

In present study, treatment protocol significantly affected pregnancy rate. In both Boran and Boran*Holstein protocols with CIDR insert (CIDR + Ovsynch & CIDR + Cosynch) yield higher pregnancy. Similar to present finding, different previous estrus cycle manipulative studies indicated an improvement of 5 to 7% pregnancy when exogenous P4 is used [25–29]. The likely reason that CIDR improved P/AI might be that it initiated cycling in acyclic cows. Also addition of CIDR to Ovsynch/ Cosynch would prevent premature occurrence of estrus before or after PGF2α and result in increased fertility [25]. CIDR also may delay the onset of ovulation in cows having spontaneous early luteolysis before the PGF2α resulting in a more synchronized ovulation. Moreover, initiation of Ovsynch protocol during the metestrus may leads to failure of the first GnRH to synchronize new follicular wave and such a failure may cause the subsequent ovulatory failure to form a subnormal CL that produces less P4 following ovulation & consequently reduced conception rate [10].

In present study insemination19h after the second GnRH (Ovsynch) resulted in higher conception rate than Cosynch. The probable reason for low pregnancy to Cosynch might be due to the early insemination that was made at 48 h of PGF2α. According to Pursley et al. [2] the lowest pregnancies were found when insemination was made at earliest (0 h) and latest time (32 h) of GnRH administration and the greatest pregnancy was when cows were inseminated at 16 h of GnRH. Although we used small sample, conception rate to Cosynch in present study was higher than previously reported by Bartolome et al. [30] and Chebel et al. [29] who reported 35.1% and 33.6%, respectively. Possibilities to the difference may be due management, and/or breed differences that affect physiological responses. Overall conception rate was not different (P > 0.05) by breed (66.7% in Boran and 53.0% in Boran*Holstein).

## Conclusions

Generally Boran cows have smaller preovulatory follicles, smaller corpus luteum, large amount of progesterone and lower rate of luteolysis to PGF2α compared to Boran*Holstein. The CL of Boran cattle seems les reactive to PGF2α than Boran*Holstein CL as the rate of luteolysis was lower in Boran than Boran*Holstein. Boran*Holstein cows have higher rate of luteolysis but lower conception rate than Boran cows. CIDR application improves P4 concentration at PGF2α and conception rate in both breed. Insemination at 19 h of GnRH had higher conception rate than insemination at GnRH administration.

## Data Availability

The data presented in this study would be available on request from the corresponding author. All data related to this study is part of a thematic research which is ongoing and due to this fact data are not publically available.

## Abbreviations

GnRH: Gonadotrophin Releasing Hormone
PGF2α: Prostaglandin F2 Alpha
P4: Plasma Progesterone
CIDR: Controlled Internal Drug Release
Ovsynch: Ovulation Synchronization
Cosynch: Ovulation Synchronization with AI at final GnRH
